# Molecular and Chemical Analysis of the Lipopolysaccharide from *Aeromonas hydrophila* Strain AH-1 (Serotype O11)

**DOI:** 10.3390/md13042233

**Published:** 2015-04-14

**Authors:** Susana Merino, Rocío Canals, Yuriy A. Knirel, Juan M. Tomás

**Affiliations:** 1 Department of Microbiology, Faculty of Biology, University of Barcelona, Diagonal 643, 08071 Barcelona, Spain; E-Mail: smerino@ub.edu; 2 Institute of Integrative Biology, University of Liverpool, Crown Street, L69 7ZB Liverpool, UK; E-Mail: rcanals@gmail.com; 3 N.D. Zelinsky Institute of Organic Chemistry, Russian Academy of Sciences, Moscow V-334, Russia; E-Mail: yknirel@gmail.com

**Keywords:** *Aeromonas*, genomics, proteomics, lipopolysaccharide, O11-antigen, LPS-core

## Abstract

A group of virulent *Aeromonas hydrophila*, *A. sobria*, and *A. veronii* biovar *sobria* strains isolated from humans and fish have been described; these strains classified to serotype O11 are serologically related by their lipopolysaccharide (LPS) *O-*antigen (*O-*polysaccharide), and the presence of an S-layer consisting of multiple copies of a crystalline surface array protein with a molecular weight of 52 kDa in the form of a crystalline surface array which lies peripheral to the cell wall. *A. hydrophila* strain AH-1 is one of them. We isolated the LPS from this strain and determined the structure of the *O-*polysaccharide, which was similar to that previously described for another strain of serotype O11. The genetics of the O11-antigen showed the genes (*wb*_O11_ cluster) in two sections separated by genes involved in biosynthesis and assembly of the S-layer. The O11-antigen LPS is an example of an ABC-2-transporter-dependent pathway for *O-*antigen heteropolysaccharide (disaccharide) assembly. The genes involved in the biosynthesis of the LPS core (*waa*_O11_ cluster) were also identified in three different chromosome regions being nearly identical to the ones described for *A. hydrophila* AH-3 (serotype O34). The genetic data and preliminary chemical analysis indicated that the LPS core for strain AH-1 is identical to the one for strain AH-3.

## 1. Introduction

Lipopolysaccharides (LPS) are amphiphilic macromolecules generally comprised of three defined regions distinguished by their genetics, structures, and function: the lipid A, the core oligosaccharide (OS) and a polysaccharide portion, *O-*antigen. While the structures of lipid A and core oligosaccharide are highly conserved among bacterial genera, the *O-*polysaccharide varies in common bacterial species [1]. In many bacteria, the *O-*antigen variations give the biological identity at an intraspecific level (serogroups, serotypes or serovars) [1]. The taxonomy of the genus *Aeromonas* is complex due to the continuous description of novel species and the rearrangement of strains and species described so far [2]. Based on 16S rRNA and 5S rRNA gene sequence comparisons, and rRNA–DNA hybridization data, Colwell *et al*. (1986) [3] proposed the creation of the family *Aeromonadaceae* including *Aeromonas* as its type and unique genus. In the current edition of *Bergey’s Manual of Systematic Bacteriology*, three genera (*Aeromonas*, *Oceanimonas* and *Tolumonas*) are listed in this family, although two more genera have been described recently, *Oceanisphaera* [4] and *Zobellella* [5].

In the latest edition of Bergey’s Manual of Systematic Bacteriology [6], 14 phenospecies that correspond to 17 genomospecies (DNA hybridization groups or HGs) were included within the genus *Aeromonas*: *A. hydrophila* (HG1), *A. bestiarum* (HG2), *A. salmonicida* (HG3), *A. caviae* (HG4), *A. media* (HG5), *A. eucrenophila* (HG6), *A. sobria* (HG7), *A. veronii* (bv. Sobria, HG8, and bv. Veronii, HG10), *A. jandaei* (HG9), *A. schubertii* (HG12), *A. trota* (HG14), *A. allosaccharophila* (HG15), *A. encheleia* (HG16), and *A. popoffii* (HG17). In recent years, 11 new species have been described; therefore, 26 species have been described in the genus *Aeomonas* until now.

The *Aeromonas* genus includes a total of 97 serotypes, serotyped from reference strains of *A. hydrophila*, *A. caviae* and *A. sobria* [7,8], although some of these strains could be misidentified because of the increasing complexity in the identification of *Aeromonas* strains at the species level [9]. However, only some of them such as O3, O6, O11, O14, O16, O18, O21, O29, O33, O34 and O41 seem to be associated with virulence for specific fish species [10] and more than 60% of the septicemia cases are related to four of these serotypes: (O11, O16, O18 and O34) [11]. Serotype O11 is associated with severe infections in humans, like septicemia, meningitis and peritonitis while serotype O34, the most common in mesophilic *Aeromonas*, is associated with wound infections in humans and outbreaks of septicemia in fishes [12].

A group of virulent *Aeromonas hydrophila*, *A. sobria*, and *A. veronii* biovar *sobria* strains isolated from humans and fish have been described; these strains are serologically related by their *O-*antigens (serotype O11), and the presence of an S-layer consisting of multiple copies of a protein (VapA) with a molecular weight of 52,000 Da in the form of a crystalline surface array peripheral to the cell wall *A. hydrophila* strain AH-1 is one of them [13]. We studied the functional genetics of the LPS-core OS and *O-*antigen of LPS from this strain, being the chemical structure of this last one similar to the one described for another strain of serotype O11 [14]. Furthermore, we found genes codifying for the production and export/assembly of the S-layer characteristic from strain AH-1, between the biosynthetic genes for O11-antigen LPS production. Genetics of the AH-1 LPS-core was also studied showing the genes involved in its biosynthesis three different chromosome regions as the ones described for *A. hydrophila* AH-3 (serotype O34) [15].

## 2. Results

### 2.1. Chemical Structure of the A. Hydrophila AH-1 O11-Polysaccharide 

**Figure 1:**
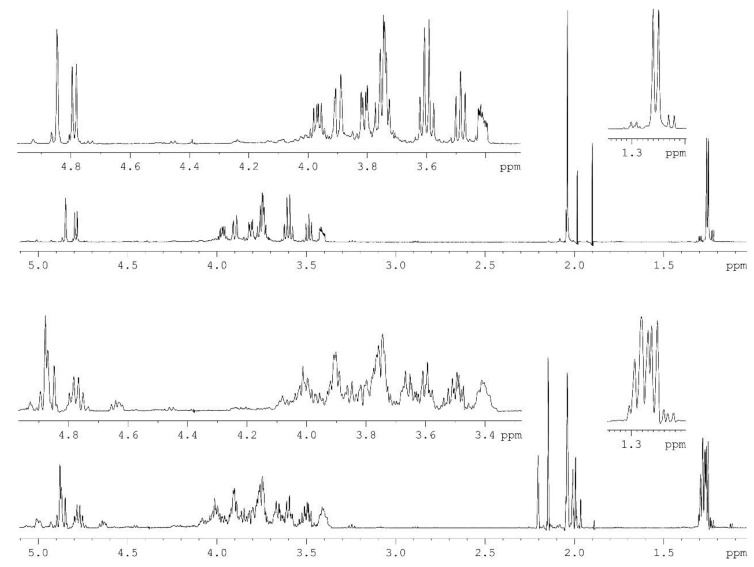
^1^H-NMR spectra of the *O-*polysaccharide (**bottom**) and *O-*deacetylated polysaccharide (**top**) from *A. hydrophila* AH-1.

**Figure 2:**
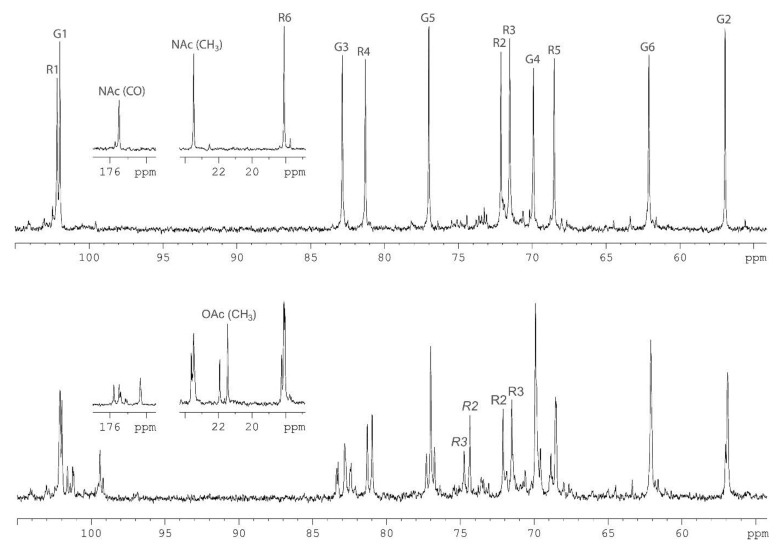
^13^С‑NMR spectra of the *O-*polysaccharide (**bottom**) and *O-*deacetylated polysaccharide (**top**) from *A. hydrophila* AH-1. Numbers refer to carbons in the GlcNAc and Rha residues denoted as G, and R, respectively. Peak annotations for the *O-*acetylated Rha residues are italicized.

The *O-*polysaccharide was obtained by mild acid degradation of the LPS isolated from bacterial cells by the Westphal procedure [16]. Sugar analysis by GLC of the acetylated alditols and (*S*)-octyl glycosides derived after full acid hydrolysis of the polysaccharide revealed l-rhamnose (l-Rha) and 2-acetamido-2-deoxy-d-glucose (d-GlcNAc). The ^1^H‑NMR (Figure 1, bottom) and ^13^C-NMR (Figure 2, bottom) spectra of the polysaccharides showed structural heterogeneity, most likely, owing to non-stoichiometric *O-*acetylation (there were signals for *O-*acetyl groups at δ_C_ 2.15and 21.9, δ_H_ 21.5 and 2.20 in the ratio ~2:1, respectively).

Indeed, spectra of the *O-*deacetylated polysaccharide (DPS) (Figure 1 and Figure 2, top) were typical of a regular polymer. They showed signals for two anomeric atoms at δ_H_ 4.78 and 4.84, δ_C_ 102.0 and 102.2, one CH_3_-C group (C6 of Rha) at δ_H_ 1.26, δ_C_ 18.1, one HO*C*H_2_-C group (C6 of GlcNAc) at δ_C_ 62.1, one nitrogen-bearing carbon (C2 of GlcNAc) at δ_C_ 56.9, other sugar protons at δ_H_ 3.42–3.97 and carbons at δ_C_ 68.5–82.9 as well as one *N*-acetyl group at δ_H_ 2.04, δ_C_ 23.5 (CH_3_) and 175.5 (CO). 

The NMR spectra of the DPS were assigned (Table 1) and the following structure of the disaccharide repeating unit of the DPS was established using two-dimensional NMR techniques essentially as described [17]: →4)-α-l-Rha*p*-(1→3)-β-d-Glc*p*NAc-(1→. 

**Table 1:** ^1^H-NMR and ^13^C-NMR chemical shifts (δ, ppm) of the *O-*deacetylated polysaccharide (DPS) from *A. hydrophila* AH-1.

Sugar Residue	Nucleus	1	2	3	4	5	6 (6a, 6b)
→3)-β-d-Glc*p*NAc-(1→	^1^H	4.78	3.76	3.61	3.49	3.42	3.74, 3.90
	^13^C	102.0	56.9	82.9	69.9	77.0	62.1
→4)-α-l-Rha*p*-(1→	^1^H	4.84	3.74	3.81	3.60	3.97	1.26
	^13^C	102.2	72.1	71.5	81.3	68.5	18.1

Chemical shifts for the *N*-acetyl group are δ_H_ 2.04, δ_C_ 23.5 (Me) and 175.5 (CO).

A comparison of the two-dimensional ^1^H, ^13^C heteronuclear single-quantum coherence (HSQC) spectra of the two polysaccharides revealed down-field displacements in both dimensions of parts of the Rha H2/C2 and H3/C3 cross-peaks from their positions in the spectrum of the DPS at δ_H_/δ_C_ 3.74/72.1 and 3.81/71.5 to δ_H_/δ_C_ 4.87/74.4 and 5.00/74.8, respectively, in the spectrum of the *O-*polysaccharide. These displacements were evidently due to a deshielding effect of the *O-*acetyl group and indicated partial *O-*acetylation of the Rha residue at position 2 or 3. As judged by relative intensities of the signals for non-acetylated and *O-*acetylated Rha residues as well as CH_3_ signals of Rha, *N*-acetyl and *O-*acetyl groups, the degree of *O-*acetylation at positions 2 and 3 is 40% and 20%, respectively. 

Therefore, the *O-*polysaccharide structure of *A. hydrophila* AH-1 (O11) may be depicted as follows:

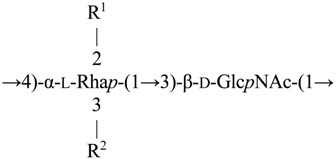


Variant I (~40%): R^1^ = R^2^ = H (non-*O-*acetylated form)

Variant II (~40%): R^1^ = Ac, R^2^ = H (major mono-*O-*acetylated form)

Variant III (~20%): R^1^ = H, R^2^ = Ac (minor mono-*O-*acetylated form)

The structure established resembles the reported *O-*polysaccharide structure of another *A. hydrophila* strain studied (SJ-44 from the collection of the Northwest Atlantic Fisheries Centre, St. John’s, Newfoundland, Canada) [14], which differs in a lower degree of *O-*acetylation (21% at position 2 of Rha and no *O-*acetyl group at position 3). Recently, and *O-*polysaccharide resembling the same structure from *A*. *sobria* strain Pt312 with a different degree of *O-*acetylation have been reported [18].

### 2.2. O11-Antigen LPS Biosynthesis Gene Cluster (wb_O11_) 

**Figure 3:**
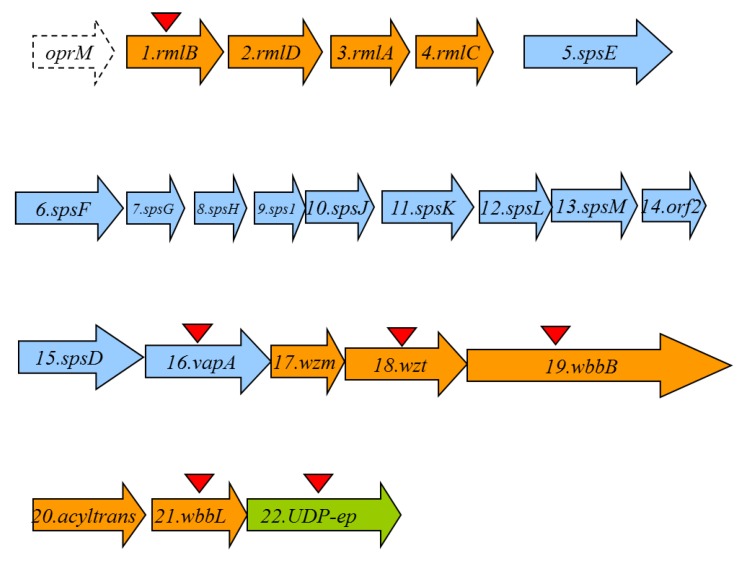
The *A. hydrophila* O11-antigen LPS (*wb*) in orange and S-layer cluster in blue open reading frames (Orfs) detected as complete genes. The genes, numbered according to the ORF number, were named according to their similarity found by their encoding proteins with proteins of well characterized functions. The *UDP-ep* gene (in green) does not belong to any of both clusters. The *oprM* gene is incompletely sequenced and should be noticed that is also adjacent to the *wb* O34-antigen LPS from strain AH-3 previously characterized [20]. Triangles indicated the in frame mutants obtained.

A cosmid-based genomic library of *A. hydrophila* AH-1 (serotype O11) was constructed and introduced into *E. coli* DH5α as previously described for other *Aeromonas* strains [19]. Because we previously detected that *Aeromonas* O11-antigen LPS contains rhamnose, we decided to construct DNA probes from *A. hydrophila* strain AH-3 *rmlA* and *B* genes (two biosynthetic rhamnose genes, [20]). We screened the *A. hydrophila* AH-1 genomic library by colony Southern blot using these two DNA probes and found several tetracycline‑resistant clones able to cross react with both probes. In order to identify the *A. hydrophila* AH-1 genes involved in the LPS O11-antigen biosynthesis, the complete nucleotide sequence of some positives recombinant clones was determined by using oligonucleotides complementary to cosmid pLA2917 [19] sequences flanking the DNA inserted. Other sequence-derived oligonucleotides were purchased (Amersham-Pharmacia Biotech) and used to complete the nucleotide sequence (GenBank KP856714). Analysis of the sequenced regions showed 22 complete putative open reading frames (ORFs) transcribed in the same direction, being nine of them (ORF1 to 4 and ORF17 to 21) genes involved in the O11-antigen LPS biosynthesis (*wb* cluster) as indicated in Figure 3 (in orange). 

Computer analysis of the *wb* gene cluster sequence revealed a conserved JUMPstart sequence with the 8 bp *ops* (operon polarity suppressor) sequence (GGCGGTAG) 132 bp upstream from the ORF1. The *ops* sequence is recognized by the bacterial antiterminator RfaH, which can be recruited by the transcription elongation complex to reduce pausing and termination at intergenic sites of polycistronic operons, allowing RNA polymerase to conclude transcription of the distal genes in large operons [21,22].

However, we found several ORFs (5–16) between the genes involved in the LPS biosynthesis that belongs to a type II secretion system (T2SS) and the structural gene *vapA* (ORF16) that corresponds to the surface S-layer protein. These genes are indicated in blue in Figure 3. It seems clear that these ORFs are genes codifying for the production and export/assembly of the S-layer characteristic from strain AH-1. It is interesting to indicate that the point of insertion of the S-layer genes seems to be a truncated gene codifying for a putative Wzm protein that could be found just upstream of the S-layer genes. Downstream of the S-layer genes it could be observed complete ORFs encoding for Wzm and Wzt proteins characteristics of an ABC-2 type transporter. Finally, we found a gene (ORF22) labeled in green in Figure 3, codifying for a NAD-dependent dehydratase or UDP-sugar epimerase (UDP-ep) that initially seems not to belong to the *wb* cluster, as described later, as their mutation do not abolish the O11-antigen LPS.

The corresponding analysis of the proteins encoded by these ORFs with their homology is presented in Table 2. All the homologies in Table 2 only included proteins with confirmed functions. 

**Table 2:** Characteristics of the *A. hydrophila O-*antigen LPS (*wb*) and S-layer cluster ORFs.

ORF	Protein Name	Protein Size	Predicted Function	Homologous Protein with Known Function	Percentage Identity/Similarity
***O-*antigen LPS (*wb*) cluster**					
1	RmlB	361	dTDP-glucose-4-6-dehydratase	RmlB multispecies *Aeromonas*	100/100
2	RmlD	295	dTDP-4-dehydro-rhamnose reductase	RmlD multispecies *Aeromonas*	100/100
3	RmlA	292	Glucose-1-phosphate thymildylyl transferase	RmlA multispecies *Aeromonas*	100/100
4	RmlC	186	dTDP-4-dehydro-rhamnose 3,5-epimerase	RmlC multispecies *Aeromonas*	93/100
	**Inserted S-layer protein cluster**				
17	Wzm	272	ABC-2 type transporter permease	Wzm multispecies *Aeromonas*	98/99
18	Wzt	438	ABC transporter ATP binding protein	Wzt multispecies *Aeromonas*	100/100
19	WbbB	1116	*N*-acetyl glucosaminyl transferase	WbbB *Klebsiella pneumonaie*	79/87
20	acyltrans	358	Acyl transferase family 3	Acyltransferase *Serratia marcescens*	45/65
21	WbbL	268	Rhamnosyl transferase	Glucosyl transferase family 2 *A. veronii* Rhmanosyl transferase *E. coli*	100/10043/67
22	UDP-ep	318	NAD-dependent dehydratase or UDP-sugar epimerase	NAD-dependent dehydratase or UDP-sugar epimerase multispecies *Aeromonas*	100/100
**S-layer protein cluster**					
5	SpsE	551	S-layer secretion system protein E	Type II secretion system (T2SS) protein E *A. hydrophila*	100/100
6	SpsF	399	S-layer secretion system protein F	Type II secretion system(T2SS) protein F *A. hydrophila*	100/100
7	SpsG	144	S-layer secretion system protein G	Type II secretion system (T2SS) protein G *A. hydrophila*	99/100
8	SpsH	123	S-layer secretion system protein H	Type II secretion system (T2SS) protein H *A. hydrophila*	98/99
9	SpsI	139	S-layer secretion system protein I	Type II secretion system (T2SS) protein I *A. hydrophila*	99/100
10	SpsJ	201	S-layer secretion system protein J	Type II secretion system (T2SS) protein J *A. hydrophila*	98/99
11	SpsK	340	S-layer secretion system protein K	Type II secretion system (T2SS) protein K *A. hydrophila*	100/100
12	SpsL	252	S-layer secretion system protein L	Type II secretion system (T2SS) protein L *A. hydrophila*	94/95
13	SpsM	198	Putative S-layer secretion system protein M	ORF1 hypothetical protein for S-layer secretion *A. hydrophila*	100/100
14	Orf2	166	Putative S-layer hypothetical protein secretion system	ORF2 hypothetical protein for S-layer secretion *A. hydrophila*	98/99
15	SpsD	743	S-layer secretion system protein D	Type II secretion system (T2SS) protein D *A. hydrophila*	100/100
16	VapA	469	Surface layer protein	Paracrystaline surface layer protein *A. hydrophila*	100/100

### 2.3. Mutant Isolation and Characterization 

**Figure 4:**
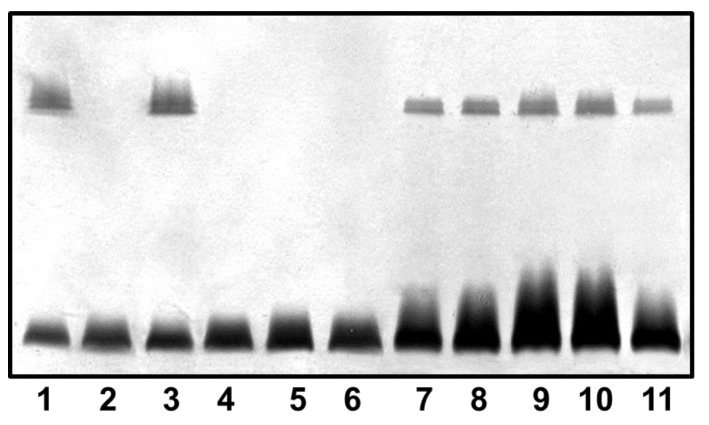
LPS analysed by SDS-PAGE (12%) and silver stained from *A. hydrophila* AH-1 (wild-type, lane 1), mutants AH-1ΔrmlB (lane 2), AH-1ΔvapA (lane 3), AH-1Δwzt (lane 4), AH-1ΔwbbB (lane 5), AH-1ΔwbbL (lane 6), AH-1ΔUDP-ep (lane 7), mutant AH-1ΔrmlB complemented with AH-1 *rmlB* (lane 8), mutant AH-1Δwzt complemented with AH-1 *wzt* (lane 9), mutant AH-1ΔwbbB complemented with AH-1 *wbbB* (lane 10), and mutant AH-1ΔwbbL complemented with AH-1 *wbbL* (lane 11).

As described in materials and methods section we obtained in frame mutants in ORFs 1, 16, 18, 19, 21, and 22 named AH-1ΔrmlB, AH-1ΔvapA, AH-1Δwzt, AH-1ΔwbbB, AH-1ΔwbbL, and AH-1ΔUDP-ep, respectively. As it can be observed in Figure 4, mutants AH-1ΔrmlB, AH-1Δwzt, AH-1ΔwbbB, and AH-1ΔwbbL were unable to produce *O-*antigen containing LPS when analyzed in a SDS-PAGE gel. However, AH-1ΔvapA and AH-1ΔUDP-ep mutants showed in the same gels identical LPS profile as the wild type strain with *O-*antigen ladder repetitions. 

**Figure 5:**
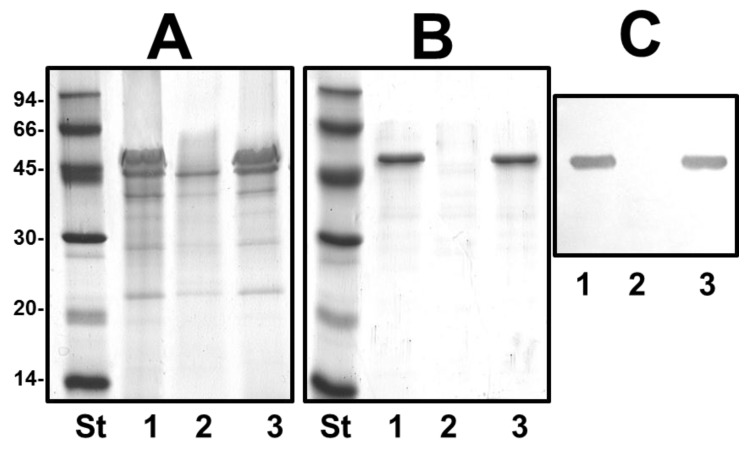
(**A**) Outer membrane proteins; (**B**) Isolated S-layer protein; and (**C**) Western blot using antiserum against S-layer protein of strains: AH-1 (wild type, lane 1), mutant AH-1ΔvapA (lane 2), and AH-1ΔvapA complemented with AH-1 *vapA* (lane 3). St, molecular weight standard.

We obtained outer-membrane proteins (OMp) and purified S-layer from mutant AH-1ΔvapA as described in materials and methods. Analysis by SDS-PAGE gels showed the lack of a protein band of approximately 52 kDa compared with the wild type (Figure 5A,B). This band reacts with specific serum anti-S protein in Western blot analysis (Figure 5C). No changes in OMp were observed for AH-1ΔUDP-ep mutant.

The reintroduction of the corresponding wild type genes in AH-1ΔrmlB, AH-1Δwzt, AH-1ΔwbbB, and AH-1ΔwbbL fully restored the LPS profile of the wild type strain in silver-stained SDS gels. A similar situation was observed when wild-type *vapA* was reintroduced in mutant AH-1ΔvapA, the presence in the OMp profile of the 52 kDa protein reacting with specific serum anti-S protein was restored (Figure 4 and Figure 5). 

**Figure 6:**
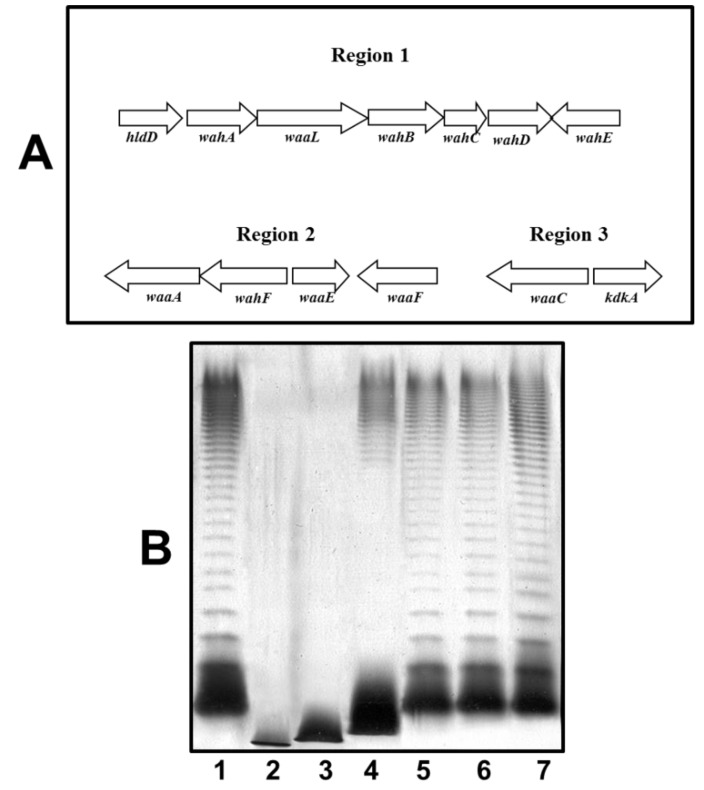
(**A**) Genetic organization of the *A. hydrophila* AH-1 three chromosomal regions containing genes for the LPS core biosynthesis. Transcription directions are indicated; (**B**) LPS analysed by SDS-PAGE (12%) and silver stained from *A. hydrophila* AH-3 serotype O34 (wild-type, lane 1), mutants AH-3ΔwaaC (lane 2), AH-3ΔwaaF (lane 3)AH-3Δ wahA (lane 4), AH-3ΔwaaC complemented with AH-1*waaC* (lane 5), AH-3ΔwaaF complemented with AH-1*waaF* (lane 6), and AH-3ΔwahA complemented with AH-1*wahA* (lane 7).

By colony Southern blot using DNA probes from the three chromosomal regions of *waa* from *A. hydrophila* strain AH-3 (*wahA* for region 1, *waaE* for region 2, and *waaC* for region 3 [15]), we screened the genomic library of *A. hydrophila* AH-1, and we were able to found several tetracycline‑resistant clones able to independently cross react with the different probes. The nucleotide sequence of the recombinant clones found for each region was determined in order to identify the *A. hydrophila* AH-1 genes conferring the LPS-core production. The complete nucleotide sequence was determined as previously indicated (GenBank KP856713). As it can be observed in Figure 6, region 1 containing seven ORFs and regions 2 and 3 consisting of 4 and 2 ORFs, respectively, showed the same organization as previously indicated for strain AH-3 [15]. 

The corresponding analysis of the proteins encoded by these ORFs from all regions renders more than 96% identity with the corresponding ones of strain AH-3. In order to confirm the gene identity of these three AH-1 regions with the corresponding ones in strain AH-3, we decided to complement three AH-3 LPS-core OS mutants (one from each region) with the corresponding AH-1 genes. Thus, AH-1 *wahA*, *waaF*, and *waaC* were independently introduced using plasmids constructed as described in materials and methods in AH-3ΔwahA, AH-3ΔwaaF, and AH-3ΔwaaC [15], respectively. The AH-3ΔwaaC and AH-3ΔwaaF mutants completely lack O34-antigen LPS and showed a faster migration of their R-form LPS in silver-stained SDS gels compared with the wild type strain. The AH-3ΔwahA mutant showed a LPS producing mainly high molecular weight O34-antigen LPS and a faster migration of their R-form LPS in gels compared with the wild type strain. When the AH-1 *wahA*, *waaF*, and *waaC* were introduced to their respective AH-3 mutants, all of them recovered identical O34-antigen LPS profiles and R-form LPS migration in gels as their wild type strain AH-3 (serotype O34), as is shown in Figure 6. No changes were observed when we introduced the plasmid vector alone.

The LPS isolated by the phenol-water procedure from the *A. hydrophila* AH-1ΔrmlB mutant (R-form LPS, as being unable to produce the *O-*antigen containing form but with a complete LPS core), was subjected to acetic acid reaction and submitted to GPC on Sephadex G-50 to render an OS mixture. This mixture showed molecular masses of 1696.8 and 1858.3 Da after ESIMS, as occurred with LPS-core of strain AH-3. GC of the alditol acetates derived after full acid hydrolysis revealed Gal, Glc, GlcN, d-*glycero*- and l-*glycero*-d-*manno*-heptose (d,dHep and l,DHep) in the ratios 0.4:1:1:2.3:4.2, respectively. Again, this analysis was completely in agreement with the one performed for OS from strain AH-3 [23].

## 3. Discussion

The *Aeromonas* O11-antigen *wb* gene cluster showed a % G+C of 48.4%, lower than the 57%–65% expected for the species of the genus *Aeromonas*, characteristic of some *wb* clusters that usually possess a G+C content at least 10% below the species average. The encoded proteins are consistent with the chemical structure of the O11-antigen LPS: RmlBDAC (ORF1-4) for the production of rhamnose, and there was no need for specific biosynthetic genes for acetyl-*N*-glucosamine (GlcNAc), and the transferases WbbL (ORF21) (rhamnosyl transferase) and WbbB-like (ORF19) (GlcNAc transferase), respectively. The ORF19 (WbbB) protein showed high homology with proteins WbbA of the *Serratia marcescens wb*_O4_ [24] and WbbB of *Klebsiella peumoniae wb*_O12_ [25]. These proteins showed two domains: a capsule polysaccharide biosynthesis protein condensation domain over the first 125–275 amino acid residues, and a glycosyltransferase family 25 (LPS biosynthesis protein) domain characteristic of GlcNAc transferases which extends from the amino acid residue 500 to the end of the protein. The last domain shared a high homology with WbbA and B proteins while the first part of the protein shared high homology but decreased with the first domain of WbbA and B proteins. All these proteins were predicted to be anchored to the membrane, and WbbA and B have been suggested to be bifunctional, being the glycosyl transferase function in common. It seems clear that ORF19 (WbbB) protein could be another one of these proteins characteristic of *O-*antigen disaccharides biosynthesized by an ABC 2 transporter-dependent pathway. There is also one putative acetyl transferase (ORF20) which is in agreement with the acyl residues found in the O11-antigen LPS chemical structure. 

ORF17 and 18 were similar to ABC-2 type transport system integral membrane and ATP-binding proteins, respectively (Table 2). Hydrophobicity analysis and identification of the putative transmembrane domains of the ORF17 protein, using the method of Klein *et al*. [26], suggest that it is an integral membrane protein. Also, the ORF18 protein showed the sequence GHNGAGKS (amino acid residues 76–83) which correspond to Walker box A, a motif present in ATP-binding proteins, as well as the ABC transporter family signature YSSGMYVRLAFAVQA (amino acid residues 148–162). Thus, ORF17 and 18 were named *wzm* and *wzt,* respectively. Two main known pathways for *O-*antigen export have been established [27], the Wzy-dependent pathway for heteropolysaccharides structures and the ABC 2 transporter-dependent pathway mainly for homopolysaccharides or disaccharides. The presence of complete Wzm (ORF17) and Wzt (ORF18) showed that the O11-antigen LPS belongs to the second pathway. To synthesize O antigens, monomers are assembled on a lipid carrier (undecaprenol phosphate) by enzymes which can be or not encoded in the *wb*_O11_ gene cluster before their incorporation into the LPS molecule. In this case, we could not find such enzyme encoded by the *wb*_O11_.

Accordingly with all these points, mutants AH-1ΔrmlB, AH-1Δwzt, AH-1ΔwbbB, and AH-1ΔwbbL were unable to produce *O-*antigen containing LPS when analyzed in an SDS-PAGE gel. However, AH-1ΔUDP-ep mutant showed an identical LPS profile as the wild type strain with the presence of *O-*antigen ladder repetitions. For this reason, we decided that ORF22 (UDP-ep) deso not belong to the *wb*_O11_ and O11-antigen LPS biosynthesis is not necessary. However, we could not exclude the remote possibility that a functional homologue could be located in a different chromosomal location.

The genes for the biosynthesis and assembly of the S-layer are inserted in the middle of the *wb* cluster (Figure 3 and Table 2). Also, the point of insertion seems to be a truncated gene codifying for a Wzm protein that could be found just upstream of the S-layer genes. Adjacent to this broken gene, it could be observed that the S-layer encoding genes included a large group of T2SS (*ORF5* to *12* and *ORF15*) genes, plus some genes encoding hypothetical proteins related to this specific S-layer T2SS system (*ORF13* and *14*), and the final gene encoding the unique protein that forms the S-layer (*vapA*, *ORF16*) characteristic of the serotype O11 strains. Downstream to them, we found the complete genes encoding for Wzm and Wzt proteins (ORF17 and ORF18, as previously indicated). According to this, the AH-1ΔvapA mutant showed identical LPS profile as the wild type strain with *O-*antigen ladder repetitions, but lacked the VapA protein being then unable to produce the S-layer.

When we inspected all the currently available *Aeromonas* genomes, we found only one that contained a copy of the *vapA* gene, the *A. veronii* Hm21 genome. Analysis of this genome shows that from ERF63505.1 to ERF63574.1, encoded products are equivalent to *A. hydrophila* AH-1 ORF1 to 22, with genes for biosynthesis and assembly of the S-layer inserted in the middle of the *wb* cluster. The point of insertion is also a truncated gene codifying for a Wzm protein. Furthermore, *A. salmonicida* is an important fish pathogen being its major virulence factor an S-layer (named A-layer), which mainly consists of a two-dimensional crystalline tetragonal proteinaceous (A-protein with a molecular weight of 49 KDa) array [28], tethered to the cell by the LPS [29]. The initial analysis of currently available genomes from this species also seems to indicate that they share the same situation as *A. hydrophila* AH-1 concerning the biosynthesis and assembly of the A-layer genes inserted in the middle of a putative *wb* cluster.

Genetics data and preliminary chemical analysis indicated that the LPS-core for strain AH-1 is identical to the one for strain AH-3. The complementation studies using AH-1 *waa* genes with AH-3 LPS-core well defined mutants are in agreement with this identity.

## 4. Materials and Methods

### 4.1. Bacterial Strains, Plasmids, and Growth Conditions

The bacterial strains and plasmids used in this study are listed in Table 3. *E. coli* strains were grown on Luria-Bertani (LB) Miller broth and LB Miller agar at 37 °C, while *A. hydrophila* strains were grown either in tryptic soy broth (TSB) or agar (TSA) at 30 °C. Ampicillin (100 µg·mL^−1^), chloramphenicol (20 µg·mL^−1^), and tetracycline (25 µg·mL^−1^) were added to the different media when required.

**Table 3:** Bacterial strains and plasmids used.

Strain or Plasmid	Relevant Characteristics	Reference or Source
***E. coli* strains**		
DH5α	F^-^*end A hsdR17* (rK^-^ mK^+^) *supE44 thi-1 recA1 gyr-A96* _*80lacZ*M15	[30]
MC1061	*thi thr1 leu6 proA2 his4 argE2 lacY1 galK2 ara14 xyl5,* *supE44*,λ*pir*	[31]
***A. hydrophila* strains**		
AH-1	O11, Wild type	[32]
AH-1ΔrmlB	AH-1 *rmlB* in frame mutant unable to produce O11-antigen LPS	This study
AH-1Δwzt	AH-1 *wzt* in frame mutant unable to produce O11-antigen LPS	This study
AH-1ΔwbbB	AH-1 *wbbB* in frame mutant unable to produce O11-antigen LPS	This study
AH-1ΔwbbL	AH-1 *wbbL* in frame mutant unable to produce O11-antigen LPS	This study
AH-1ΔUDP-ep	AH-1 *UDP-ep* in frame mutant, able to produce O11-antigen LPS	This study
AH-1ΔvapA	AH-1 *vapA* in frame mutant, unable to produce S-layer but able to produce O11-antigen LPS	This study
AH-3ΔwahA	AH-3 *wahA* LPS-core in frame mutant	[15]
AH-3ΔwaaF	AH-3 *waaF* LPS-core in frame mutant	[15]
AH-3ΔwaaC	AH-3 *waaC* LPS-core in frame mutant	[15]
**Plasmids**		
pGEMT easy	PCR generated DNA fragment cloning vector Amp ^R^	Promega
pBAD33-Cm	Arabinose-inducible expression vector, Cm ^R^	[33]
pDM4	Suicide plasmid, Sacarose, Cm ^R^	[31]
pLA2917	Cosmid vector, Km ^R^, Tc ^R^	[19]

^R^ resistant.

### 4.2. General DNA Methods

General DNA manipulations were done essentially as previously described [34]. DNA restriction endonucleases, T4 DNA ligase, *E. coli* DNA polymerase (Klenow fragment), and alkaline phosphatase were used as recommended by the Sigma-Aldrich (St Louis, MI, USA). 

### 4.3. DNA Sequencing and Computer Analysis of Sequence Data

Double-stranded DNA sequencing was performed by using the dideoxy-chain termination method [35] from PCR amplified DNA fragments with the ABI Prism dye terminator cycle sequencing kit (PerkinElmer, Barcelona, Spain). Oligonucleotides used for genomic DNA amplifications and DNA sequencing were purchased from Sigma-Aldrich (St Louis, MI, USA). Deduced amino acid sequences were compared with those of DNA translated in all six frames from nonredundant GenBank and EMBL databases by using the BLAST [36] network service at the National Center for Biotechnology Information and the European Biotechnology Information. ClustalW was used for multiple-sequence alignments [37].

### 4.4. Mutant Construction 

The chromosomal in-frame AH-1ΔrmlB, AH-1ΔvapA, AH-1Δwzt, AH-1ΔwbbB, AH-1ΔwbbL, and AH-1ΔUDP-ep deletion mutants were constructed by allelic exchange as described by Milton *et al*. [31]. Plasmids were transferred to *A. hydrophila* strains as previously indicated [20]. To complete the allelic exchange, the integrated suicide plasmid was forced to recombine out of the chromosome by growing on agar plates containing 10% sucrose. Mutants were selected based on surviving on plates containing 10% sucrose and loss of the chloramphenicol resistant marker of vector pDM4. The mutations were confirmed by sequencing of the whole constructs in amplified PCR products.

### 4.5. Plasmid Constructions and Mutant Complementation Studies 

For complementation studies, the *A. hydrophila* AH-1 *rmlB*, *vapA*, *wzt*, *wbbB*, *wbbL*, *wahA*, *waaF*, and *waaC* genes were PCR amplified using appropriate oligonucleotides obtained from the sequenced clones and chromosomal AH-1 DNA as template, ligated to plasmid pGEMT (Promega), and transformed into *E. coli* DH5α. After checked, the corresponding genes were subcloned on plasmid pBAD33-Cm with an arabinose-inducible and glucose-repressible promoter [33]. Induction was obtained by adding l-arabinose to a final concentration of 0.2% (*w*/*v*). Plasmids were transferred to *A. hydrophila* strains as previously indicated [20].

### 4.6. LPS Isolation and SDS-PAGE

For screening purposes, LPS was obtained after proteinase K digestion of whole cells [37]. LPS samples were separated by sodium dodecyl sulfate-polyacrylamide gel electrophoresis (SDS-PAGE) and visualized by silver staining as previously described [38].

### 4.7. Large-Scale Isolation of LPS Plus Isolation and O-Deacetylation of the O-Polysaccharide

Cells (3 g dried weight) were digested with DNase, RNase (24 h, 3 mg each) and Proteinase K (36 h, 3 mg) in 25 mM Tris-HCl buffer pH 7.63 containing 2 mM CaCl_2_ (30 mL), the suspension was dialysed against distilled water and freeze-dried. Digested cells were extracted with aqueous 45% phenol at 68 °C [16], the extract was dialysed against tap water without separation of the layers, residual cells were removed by centrifugation, and the supernatant was freeze-dried to give lipopolysaccharide in a yield of ~8%. A lipopolysaccharide sample (120 mg) was degraded with 0.1 M sodium acetate buffer pH 4.2 for 4 h at 100 °C, the lipid precipitate was removed by centrifugation (13,000× *g*, 20 min), and a high-molecular-mass *O-*polysaccharide (34 mg) was isolated from the supernatant by gel-permeation chromatography on a column (50 × 2.5 cm) of Sephadex G-50 Superfine in pyridinium acetate buffer (4 mL pyridine and 10 mL HOAc in 1 L water) using a Knauer differential refractometer (Berlin, Germany) for monitoring. *O-*Deacetylation of the polysaccharide was performed by heating with aqueous 12% ammonia (2 mL) for 3 h at 60 °C, ammonia was removed by stream of air, and the remaining solution was freeze-dried. 

### 4.8. Sugar Analysis and NMR Spectroscopy

For sugar analysis, a polysaccharide sample (0.5 mg) was hydrolyzed with 2 M CF_3_CO_2_H (100 °C, 4 h), the monosaccharides were conventionally converted into the alditol acetates [39] and analyzed by GLC on a Varian 3700 chromatograph (Santa Clara, CA, USA) equipped with a fused-silica gel SPB-5 column using a temperature gradient from 150 °C (3 min) to 320 °C at 5 °C min^−1^. The absolute configurations of the monosaccharides were determined as described [40], using the same GLC conditions as in sugar analysis.

NMR spectra were obtained on a Bruker DRX-600 spectrometer (Bremen, Germany) in 99.96% D_2_O at 60 °C using internal acetone (δ_H_ 2.225, δ_C_ 31.45) as calibration reference. Prior to the measurements, the samples were lyophilized twice from 99.9% D_2_O. Bruker XWINNMR 1.3 software was used to acquire and process the data. A mixing time of 100 and 200 ms was used in two-dimensional TOCSY and ROESY experiments, respectively.

### 4.9. OM Protein and S-Layer Isolation and Characterization

Outer membranes (OM) were obtained by incubating membrane suspensions with 3% Sarkosyl in 20 mM TrisHCl buffer (pH 8.0) for 20 min at room temperature, as previously described [41]. The S-layer sheet material was obtained by using a modification of the procedure of Dooley and Trust [42]. Cells were grown overnight in 1000 mL of Luria Broth (LB) with agitation (200 rpm), harvested by centrifugation (12,000× *g*, 20 min), and washed twice in 20 mM Tris-HCl (pH 8.0). They were suspended in 100 mL of 0.2 M glycine-HCl (pH 2.8) and stirred at 4 °C for 30 min. The cells were removed by a single centrifugation at 12,000× *g* for 20 min. The S-layer sheet material was collected by centrifugation at 40,000× *g* for 60 min, suspended in 500 μL of 20 mM Tris-HCl (pH 8.0), and frozen at −20 °C.

Protein were analysed by SDS-PAGE and separated protein bands were visualized by Coomassie Brilliant blue staining as previously described [41]. Anti-purified-S-layer antiserum was obtained and assayed as previously described [43]. After SDS-PAGE, immunobloting was carried out as previously described [44].
